# Are rural South African children abdominally obese?

**DOI:** 10.12669/pjms.293.3136

**Published:** 2013

**Authors:** AL Toriola, VK Moselakgomo, BS Shaw, DT Goon, FC Anyanwu

**Affiliations:** 1 AL Toriola, Department of Sports, Rehabilitation and Dental Sciences, Tshwane University of Technology, Pretoria, South Africa.; 2 VK Moselakgomo, Department of Sports, Rehabilitation and Dental Sciences, Tshwane University of Technology, Pretoria, South Africa.; 3 BS Shaw, Department of Sport and Movement Studies, University of Johannesburg, PO Box 524, Auckland Park, Johannesburg, 2006, Republic of South Africa.; 4 DT Goon, Centre for Biokinetics, Recreation and Sports Science, University of Venda, Thohoyandou, South Africa.; 5 FC Anyanwu, Centre for Biokinetics, Recreation and Sports Science, University of Venda, Thohoyandou, South Africa.

**Keywords:** Abdominal obesity, Waist-to-height ratio, Rural children

## Abstract

***Objectives:*** While available data exist on total body fat of rural South African children, as measured by body mass index, little is known concerning the abdominal obesity of rural South African children. The aim of this study was to determine the prevalence of abdominal obesity among rural South African children.

***Methods*** **:** Participants involved 1 172 rural black school children (541 boys and 631 girls) aged 10−16 years, residing in Mankweng and Toronto, both rural black settlements in Capricorn district, Limpopo province, South Africa. Height, weight and waist circumference were measured using standard techniques. Waist-to-height ratio (WHtR) was calculated. A WHtR ≤ 0.50 was used to determine abdominal obesity. Results were analysed using student t-test and Chi-squared statistics, with a p-value of < 0.05.

***Results*** **:** Waist-to-height ratio showed inconsistent results in both sexes and across age groups, with no significant differences among boys and girls in all age groups. The proportion of boys with a WHtR ≥ 0.5 was 69 (12.8%), while girls were 92 (14.6%). The highest proportion of WHtR occurs at age 11 in boys, while this proportionality increases with age in girls, peaking at ages 14-16 years. Overall, 161 (13.7%) children had central obesity.

***Conclusions*** **:** This study indicates that abdominal obesity as measured by WHtR is prevalent among rural black South African children. The prevalence of WHtR ≥ 0.5 (13.7%) among the children is worrisome, as its signals the presence of obesity-related problems and the likely susceptibility of these sample children to future health risks. Therefore, interventions strategies are needed to reduce central obesity among children.

## INTRODUCTION

The importance of total body fat and distribution is a major risk factor for both adults and children.^1^ It is reported that the incidence of diabetes, atherosclerosis and sudden cardiac death is high among obese persons, but metabolic and cardiovascular adverse effects are even more significant when obesity is centered around the abdomen.^2^ Therefore, early identification and treatment of children with central obesity is essential in order to plan appropriate remedial strategies for their health. Thus, information on abdominal obesity is relevant judging from both an epidemiological and clinical viewpoint.

Several studies^3^^-^^7^ have reported a high prevalence of overweight and obesity among South African children. A meta-analytical study^8^ investigating the prevalence and intrinsic patterns of childhood overweight and obesity in South Africa covering 1998-2008, indicated that the high levels of overweight and obesity among South African children, occurs predominantly in the urban settings. The review also indicated that the trend in the prevalence of overweight and obesity in South African children is patterned along gender, race, age and socio-demographic and socio-economic factors.

Some studies^3^^-^^5^ have confirmed the occurrence of total obesity as measured by BMI among rural South African children. However, little is known concerning the prevalence of abdominal obesity among rural South African children. Only few studies^6^^,^^9^^,^^10^ have assessed the prevalence of abdominal obesity among South African children, especially among rural children. As such, whether abdominal obesity is prevalent among rural South African children is only speculative.

Although other precise methods of measuring abdominal obesity such as computed tomography, nuclear magnetic resonance imaging are available, few population studies using these methods exists, primarily due to its high operational cost. In this regards, anthropometric measures appears to be a good alternative for the diagnosis of abdominal obesity.^11^ Therefore, the purpose of this study was to assess abdominal obesity in rural South African residing in Mankweng and Toronto, Limpopo province, using the anthropometric index of waist-height-ratio (WHtR). This anthropometric index has been used by several researchers to evaluate abdominal obesity among children^12^^-^^17^ and is known to correlate well with cardiovascular disease risk factors in children and adolescents.^17^^-^^19^

The present study was designed to screen for abdominal obesity among rural South African children in Mankweng and Toronto regions, using WHtR.

## METHODS

A stratified random sampling of 1172 (541 boys and 631 girls) children aged 10-16 years, attending seven primary schools in Mankweng and Toronto, both rural settlements in Capricorn district, Limpopo province, South Africa, participated in the study. Procedures used for sampling and collection of anthropometric data have been reported in detail elsewhere.^5^ The Central Higher Degrees Committee of Tshwane University of Technology, Pretoria, South Africa, and other relevant provincial regulatory authorities granted ethics approval for the research to be carried out. An information leaflet and informed consent form were administered to the head teachers, pupils and their parents or guardians who consented that the study be carried out.

Height, weight and waist circumference were measured using standard techniques.^20^ Waist-to-height ratio was calculated by dividing waist circumference (in cm) by stature (in cm). A WHtR ≤ 0.50 was used to determine abdominal obesity.^21^ Results were analysed using student t-test and Chi-squared statistics, with a p-value of < 0.05.

## RESULTS

Shown in Table-I are the anthropometric measurements of the participants stratified by gender. The mean age of the participants was 12.3 ± 1.2 (boys 12.5 ± 1.3; girls 12.2 ± 1.2). Girls had significantly higher mean values in all the anthropometric variables compared to boys. A total of 161 (13.7%) children had central obesity as measured by WHtR. There was no significant (p = 0.157; p ≥ 0.05) difference in the mean value of WHtR between boys and girls. Waist-to-height ratio showed inconsistent results in both sexes and across age groups, with no significant differences among boys and girls in all age groups (Table-II). The proportion of boys with a WHtR ≥ 0.5 was 69 (12.8%), while girls were 92 (14.6%). The highest proportion of WHtR occurs at age 11 in boys, while this proportionality increases with age in girls, peaking at ages 14-16 years (Table-III).

**Table-I:** Anthropometric measurements of the participants according to gender

	*Boys (n = 541)*	*Girls (n =631)*	*Combined (n = 1172)*	*p-value*
*Variables *	*Mean ± SD*	*Mean ± SD*	*Mean ± SD*	
Height (cm)	140.5 ± 13.6	143.2 ± 12.2	141.9 ± 12.9	0.001*
Body weight (kg)	40.5 ± 9.9	43.6 ± 10.8	42.1 ±10.5	0.001*
Waist (minimum) (cm)	59.8 ± 9.0	61.9 ± 9.5	7.0 ± 6.1	0.001*
Waist-to-height ratio	0.45 ± 0.1	0.47 ± 0.1	0.44 ± 0.1	0.002*

*Statistically significant (p ≤ 0.05)

**Table-II:** Waist-to-height ratio of South African children according to age groups

			*Waist-to-height ratio*		
*Age (years)*	*Boys *	*Girls *	*Boys (Mean ± SD)*	*Girls (Mean ± SD)*	*p-value*
10	26	42	0.43±0.05	0.43±0.07	0.773
11	110	147	0.44±0.07	0.43±0.08	0.463
12	150	162	0.43±0.06	0.44±0.06	0.588
13	133	194	0.42±0.07	0.44±0.06	0.095
14-16	122	86	0.42±0.06	0.43±0.07	0.165
Total	541	631	0.43±0.06	0.43±0.01	0.157

*Statistically significant (p ≤ 0.05); CI = Confidence Interval

**Table-III:** Percentage of children with a WHtR at 0.5 or above cut-off point

		*WHtR ≥0.5*
*Age (years)*	*n *	*%*
Boys		
10	4	15.4
11	20	18.2
12	25	16.7
13	14	10.5
14-16	6	4.9
Total	69	12.8
Girls		
10	5	11.9
11	19	12.9
12	26	16.0
13	31	16.0
14-16	11	12.8
Total	92	14.6
Boys/ Girls	161	13.7

n = number of sample;    % = percentage;    WHtR = waist-to-height ratio

## DISCUSSION

The WHtR is a valid method for assessing excessive amount of upper body fat that poses a risk to health^22^ with a simple message: keep your waist circumference to less than half your height’.^15^ This anthropometric index is easy and inexpensive can be used in laboratory and field settings. The WHtR indicator assumes that individuals with a certain anthropometric measure of fat patterning would have the same degree of fat regardless of their age, race, or gender. Also, WHtR has the advantage of better measurement of fat distribution in different ages and statures.^23^ This anthropometric index is not extensively studied in South African children, especially in rural children. 

This study shows that the proportion of children with a WHtR ≥ 0.5 exists in both genders and in all age groups. The proportionality of WHtR ≥ 0.5 is higher in girls (14.6%) compared to boys (12.8%). Similarly, among one to 20 years black South African rural children, 10% of adolescents are reported to have WHtR ≥ 0.5, with a significantly higher proportion of girls (15.0%) than boys (3.0%).^23^ In Naude, Kruger and Pienaar’s^24^ study involving black South African adolescents, girls (3.5%) had WHtR ≥ 0.5, whereas none of the boys exhibited a WHtR ≥ 0.5. The figures of WHtR ≥ 0.5 (13.7%) found among rural children in these regions is unexpected and disturbing from a rural setting where lifestyles and other life habits are supposed to be different from those of urban setting. Decreased physical activities, sedentary lifestyles, altered eating patterns, and increased fat content of the diet among the children could be possible reasons for their high fat deposition. However, the present study did not evaluate these factors, which would have permitted further inquiry in this regard. Notwithstanding, the present findings appear to suggest that rural life does not necessarily encompass physically demanding tasks anymore^25^ and may be a factor in the prevalence of abdominal obesity in rural areas. Disturbingly, abdominal obesity is known to be associated with cardiovascular and metabolic^2^^,^^7^^,^^18^^,^^19^ disease risk factors in children and adolescents. Therefore, the future health of the children might be jeopardised. This indicates the need for interventions to reduce central obesity in all school populations and intensive community-based efforts to prevent it beginning very early in life.

The results of this study need to be interpreted with the understanding that the study is limited in several aspects. It is appropriate to assume that the sampled population represents the adolescent children in Mankweng and Toronto, but far from being a good sample at the provincial or the national level. Given the area and size of the sample, one must be very careful in generalising the findings obtained in this study. Also, the cross-sectional nature of the study limits inferences about causality and its direction.^5^

Ideally, abdominal obesity could be assessed using criterion measures such as computed tomography, nuclear magnetic resonance imaging. However, the use of these methods has limited applicability when screening larger sample sizes such as this, especially in developing countries like South Africa, largely because it is expensive, unavailable and time-consuming.^6^ In its place, measurements of anthropometry (height and waist circumference) were taken to screen for central fat pattern in group of South African rural children living in Mankweng and Toronto regions.

The strength of this study is based on the fact that it was conducted using a fairly large sample of rural black school children. This therefore, provides the opportunity to collect information in an understudied region that can be compared with results of very few previous studies carried out in South Africa concerning abdominal obesity using the same anthropometric index^6^^,^^23^^,^^24^ although with varying methodological and geographical settings.

## CONCLUSION

The present study did not seek to determine the aetiology of abdominal obesity development in children, but to examine the prevalence of abdominal obesity among the children using the WHtR index. The analysis indicate that abdominal obesity is prevalent among rural black South African children in Mankweng and Toronto settlements. The prevalence of WHtR ≥ 0.5 (13.7%) among the children is worrisome, and typifies the presence of obesity-related problems and indicates the possibility of the children been susceptible to future health risks. Therefore, interventions strategies are warranted to reduce central obesity among children.
